# Supplementary material for: Wrinkling on Stimuli-Responsive Functional Polymer Surfaces as a Promising Strategy for the Preparation of Effective Antibacterial/Antibiofouling Surfaces
Source: Polymers (Basel). 2021 Dec 6;13(23):4262. doi: 10.3390/polym13234262 (PMC8659726; doi:10.3390/polym13234262)
Supplement: Supplementary file 1 [file polymers-13-04262-s001.zip › polymers-1438418-supplementary.pdf]

Supplementary Material.

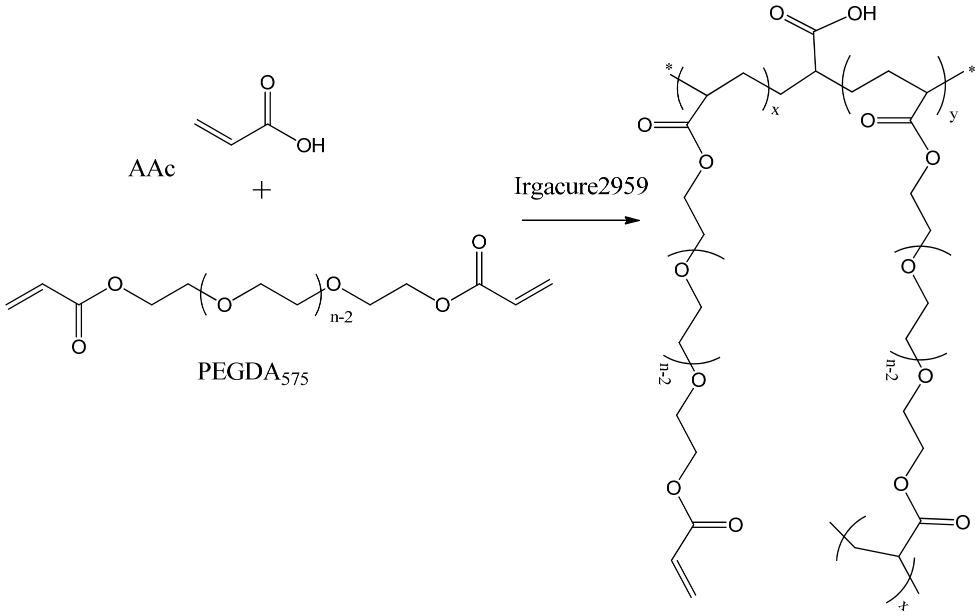

Figure S1. Schematic representation of the proposed chemical structure of *net-poly*(AAc-co-PEGDA575).

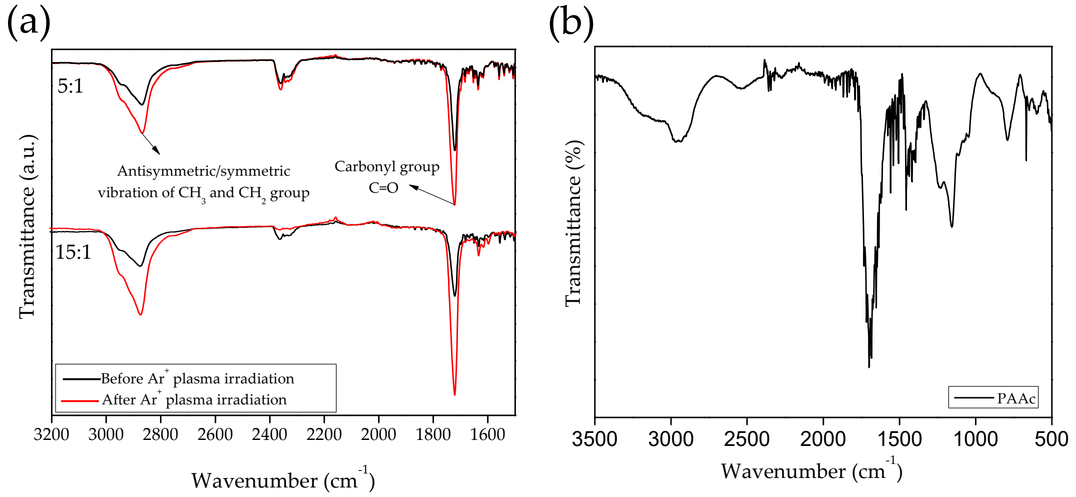

Figure S2. ATR-FTIR spectra of a) *net-poly*(AAc-co-PEGDA575) with a mole ratio of 5:1 and 15:1 before and after argon plasma irradiation and b) polyAAc (PAAc).

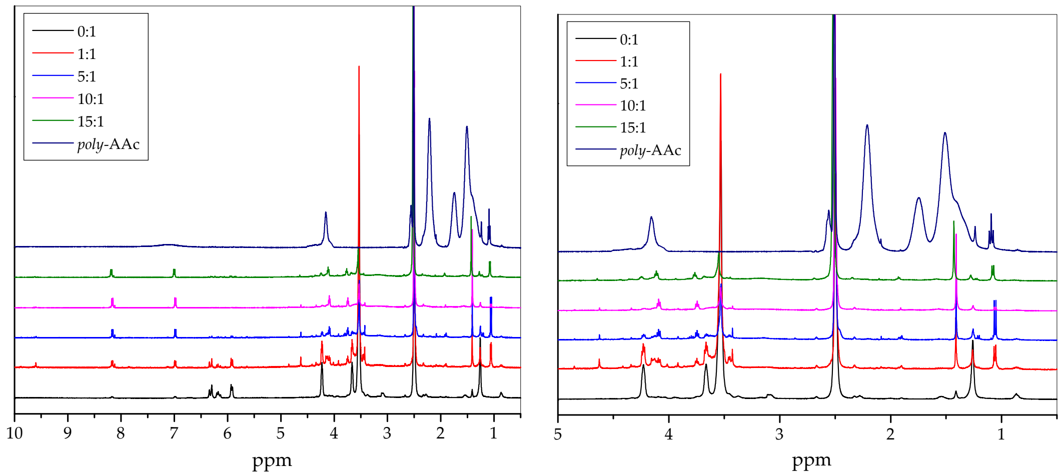

Figure S3. ^{1}H-NMR spectra of *net-poly*(AAc-*co*-PEGDA$_{575}$) at different mole ratio from 0:1 to 15:1 and *poly*-AAc.

Description of signals identified by nuclear magnetic resonance (NMR)

net-poly(AAc-*co*-PEGDA$_{575}$) at 0:1 mole ratio.

^{1}H-NMR (DMSO-d$_6$): 6.34 and 6.30 (*trans* **CH$_2$**=CH-C(O)-OR, **1.46H**, d); 6.21 and 6.16 (CH$_2$=**CH**-C(O)-OR, **1.0H**, t); 5.93 and 5.91 (*cis* **CH$_2$**=CH-C(O)-OR, **1.57H**, d); 4.24-4.22 (CH$_2$=CH-C(O)-O-**CH$_2$**-, **3.92H,** s); 3.66 (-CH$_2$-CH-C(O)-O-CH$_2$-**CH$_2$**-, **4.38H**, t); 3.53(-(**CH$_2$**-**CH$_2$**-O)$_8$-, **25.42H, s**); 2.67 (-CH$_2$-**CH**(R)$_n$-, **0.28H**, s); 2.33 (-**CH**(R)-CH$_2$-CH$_2$C(O)-OR-, **0.79H**, t); 1.90 ((-**CH$_2$**-CH(R)$_n$-, **0.17H**, s); 1.41 (-(CH$_2$-**CH**(R)$_n$-CH$_2$-), **0.63H,** s); 1.26 (-(CH$_2$-CH(R))$_{x,y}$**CH$_3$***, **5.70H**, d).

net-poly(AAc-*co*-PEGDA$_{575}$) at 1:1 mole ratio.

^{1}H-NMR (DMSO-d$_6$): 9.60 (CH$_2$=CH-COO**H**, **1.32H**, s); 6.34 and 6.30 (*trans* **CH$_2$**=CH-C(O)-OR, **3.69H,** d); 6.21 and 6.16 (CH$_2$=**CH**-C(O)-OR, **1.0H**, t); 6.11 and 6.04 (CH$_2$=**CH**-C(O)-OH, **0.09H**, s); 5.94 and 5.91 (*cis* **CH$_2$**=CH-C(O)-OR, **4.48H**, d); 4.24-4.22 (CH$_2$=CH-C(O)-O-**CH$_2$**-, **14.74H,** t); 4.10-4.08 ((-**CH**(COOH)-CH$_2$)$_n$-, **6.91H**, m); 3.76-3.73 (-**CH$_2$**-CH(COOH)-CH$_2$-, **5.82H**, t); 3.68-3.63(-CH$_2$-**CH$_2$**-

C(O)-O-CH$_2$-, **16.18H, m**); 3.53 (-(C**H**$_2$-C**H**$_2$-O)$_8$-,**126.58H**, s); 2.67 (-CH$_2$-C**H**(R)$_n$-, **1.68H**, s); 2.33 (-C**H**(R)-CH$_2$-CH$_2$C(O)-OR-, **2.20H**, s); 1.90 (-C**H**$_2$-CH(R)$_n$-, **1.56H**, s); 1.41 (-(CH$_2$-C**H**(R)$_n$-CH$_2$-), **11.62H,** s); 1.26 (-(CH$_2$-CH(R))$_{x,y}$C**H**$_3$*,**9.89H**, d) and 1.07-1.05 (-(CH(COOH)-C**H**$_3$*)$_n$-, **9.40H**, d).

net-poly(AAc-*co*-PEGDA$_{575}$) at 5:1 mole ratio.

^{1}H-NMR (DMSO-d$_6$): 9.60 (CH$_2$=CH-COO**H**, **1.55H**, s); 6.34 and 6.30 (*trans* C**H**$_2$=CH-C(O)-OR, **2.71H**, d); 6.21 and 6.16 (CH$_2$=C**H**-C(O)-OR, **1.0H**, t); 6.11 and 6.04 (CH$_2$=C**H**-C(O)-OH, **2.38H**, s); 5.94 and 5.91 (*cis* C**H**$_2$=CH-C(O)-OR, **3.89H**, d); 4.24-4.22 (CH$_2$=CH-C(O)-O-C**H**$_2$-, **17.40H,** t); 4.10-4.08 ((-C**H**(COOH)-CH$_2$)$_n$-, **24.82H**, m); 3.76-3.73 (-C**H**$_2$-CH(COOH)-CH$_2$-, **19.22H**, t); 3.68-3.63(-CH$_2$-C**H**$_2$-C(O)-O-CH$_2$-, **20.0H**, m); 3.53 (-(C**H**$_2$-C**H**$_2$-O)$_8$-,**122.01H**, s); 2.67 (-CH$_2$-C**H**(R)$_n$-, **6.35H**, s); 2.33 (-C**H**(R)-CH$_2$-CH$_2$C(O)-OR-, **6.41H**, s); 1.90 (-C**H**$_2$-CH(R)$_n$-, **12.69H**, s); 1.41 (-(CH$_2$-C**H**(R)$_n$-CH$_2$-), **48.44H,** s); 1.26 (-(CH$_2$-CH(R))$_{x,y}$C**H**$_3$*, **23.0H**, d) and 1.07-1.05 (-(CH(COOH)-C**H**$_3$*)$_n$-, **40.77H**, d).

net-poly(AAc-*co*-PEGDA$_{575}$) at 10:1 mole ratio.

^{1}H-NMR (DMSO-d$_6$): 9.60 (CH$_2$=CH-COO**H**, **8.69H**, s); 6.34 and 6.30 (*trans* C**H**$_2$=CH-C(O)-OR, **1.28H**, d); 6.26 (trans (C**H**$_2$=CH-C(O)-OH, **0.61H**, d); 6.21 and 6.14 (CH$_2$=C**H**-C(O)-OR, **1.0H**, t); 6.11 and 6.04 (CH$_2$=C**H**-C(O)-OH, **0.41H**, s); 5.94 and 5.91 (*cis* C**H**$_2$=CH-C(O)-OR, **3.11H**, d); 5.83-5.85 (C**H**$_2$=CH-C(O)-OH, **1.69H**, d); 4.24-4.22 (CH$_2$=CH-C(O)-O-C**H**$_2$-, **48.57H,** t); 4.10-4.08 ((-C**H**(COOH)-CH$_2$)$_n$-, **127.97H**, m); 3.76-3.73 (-C**H**$_2$-CH(COOH)-CH$_2$-, **114.59H**, t); 3.68-3.63(-CH$_2$-C**H**$_2$-C(O)-O-CH$_2$-, **12.00H**, m); 3.53 (-(C**H**$_2$-C**H**$_2$-O)$_8$-, **331.79H**, s); 2.67 (-CH$_2$-C**H**(R)$_n$-, **27.35H**, s); 2.33 (-C**H**(R)-CH$_2$-CH$_2$C(O)-OR-, **42.97H**, s); 1.90 (-C**H**$_2$-CH(R)$_n$-, **16.28H**, s); 1.41 (-(CH$_2$-C**H**(R)$_n$-CH$_2$-), **343.56H,** s); 1.26 (-(CH$_2$-CH(R))$_{x,y}$C**H**$_3$*, **67.70H**, d) and 1.07-1.05 (-(CH(COOH)-C**H**$_3$*)$_n$-, **31.49H**, d).

net-poly(AAc-*co*-PEGDA$_{575}$) at 15:1 mole ratio.

^{1}H-NMR (DMSO-d$_6$): 9.60 (CH$_2$=CH-COO**H**, **1.17H**, s); 6.34 and 6.30 (*trans* C**H**$_2$=CH-C(O)-OR, **1.16H**, d); 6.26 (trans (C**H**$_2$=CH-C(O)-OH, **0.56H**, d); 6.21 and 6.14 (CH$_2$=C**H**-C(O)-OR, **1.0H**, t); 6.11 and 6.04 (CH$_2$=C**H**-C(O)-OH, **0.87H**, s); 5.94 and 5.91 (*cis* C**H**$_2$=CH-C(O)-OR, **1.61H**, d); 5.83-5.85 (C**H**$_2$=CH-C(O)-OH, **1.02H**, d); 4.24-4.22 (CH$_2$=CH-C(O)-O-C**H**$_2$-, **7.88H,** t); 4.10-4.08 ((-C**H**(COOH)-CH$_2$)$_n$-, **14.35H**, m); 3.76-3.73 (-C**H**$_2$-CH(COOH)-CH$_2$-, **11.87H**, t); 3.68-3.63 (-CH$_2$-C**H**$_2$-C(O)-O-CH$_2$-, **8.33H, m**); 3.53 (-(C**H**$_2$-C**H**$_2$-O)$_8$-, **39.87H**, s); 2.67 (-CH$_2$-C**H**(R)$_n$-, **3.80H**, s); 2.33 (-C**H**(R)-CH$_2$-CH$_2$C(O)-OR-, **5.08H**, s); 1.90 (-C**H**$_2$-CH(R)$_n$-, **6.58H**, s); 1.41 (-(CH$_2$-C**H**(R)$_n$-CH$_2$-), **37.66H,** s); 1.26 (-(CH$_2$-CH(R))$_{x,y}$C**H**$_3$*, **9.59H**, d) and 1.07-1.05 (-(CH(COOH)-C**H**$_3$*)$_n$-, **17.83H**, d).

Poly(AAc)

^{1}H-NMR (DMSO-d$_6$): ^{1}H-NMR (DMSO-D$_6$): 7.09 (CH-CH-COO**H**, **3H**, s); 2.58-2.56 (-C**H**(COOH)-CH$_2$-)$_n$, **40H**, t); 2.21-1.24 (-CH(COOH)-C**H**$_2$-)$_n$, **80H**, m) and 1.11-1.08 (-(CH(COOH)-C**H**$_3$*)$_n$, **3H**, t).

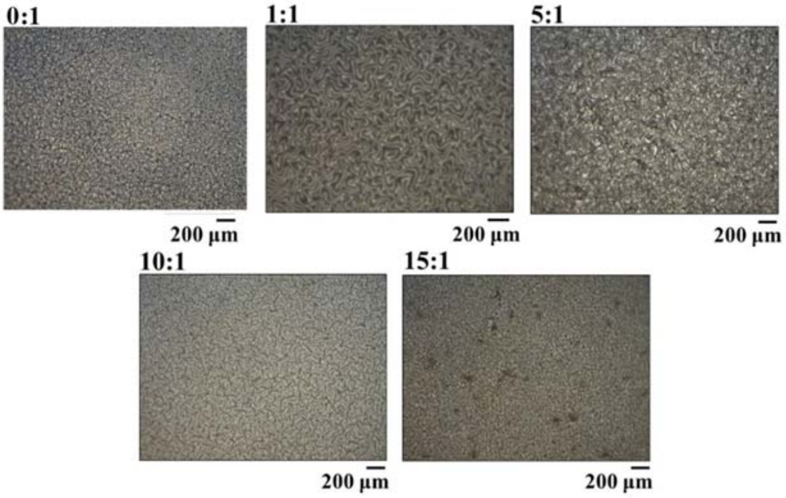

Figure S4. Optical microscopy images of the samples AAc:PEGDA$_{575}$ at different mole ratios.
